# Interleukin-13 inhibits cytokines synthesis by blocking nuclear factor-κB and c-Jun N-terminal kinase in human mesangial cells^☆^

**DOI:** 10.1016/S1674-8301(10)60043-7

**Published:** 2010-07

**Authors:** Chunhua Zhu, Aihua Zhang, Songming Huang, Guixia Ding, Xiaoqin Pan, Ronghua Chen

**Affiliations:** a Nanjing Children's Hospital Affiliated to Nanjing Medical University, Nanjing 210008, Jiangsu Province, China; b Institute of Pediatrics, Nanjing Medical University, Nanjing 210029, Jiangsu Province, China

**Keywords:** mesangial cells, interleukin-13, inflammation

## Abstract

**Objective:**

Monocytes/macrophages, proinflammatory cytokines and chemokines are important in the pathogenesis of glomerulonephritis. Interleukin (IL) -13 has been shown to exert potent anti-inflammatory properties. This study was designed to investigate the effect of IL-13 on the expression of proinflammatory cytokines, chemokines and profibrogenic cytokines and the involved molecular mechanism in cultured human mesangial cells (HMCs).

**Methods:**

The expressions of proinflammatory cytokines, chemokines and profibrogenic cytokines were determined by ribonuclease protection assay (RPA). Activity of nuclear factor-kappa B (NF-κB) and activator protein-1 (AP-1) was examined by electrophoretic mobility shift assay (EMSA). NF-κB subunit p65 nuclear transportation and c-Jun N-terminal kinase (JNK) activity were assayed by immunoblot.

**Results:**

Recombinant IL-13 inhibited tumor necrosis factor-α (TNF-α), IL-1α, IL-1β, monocyte chemoattractant protein-1 (MCP-1), IL-8, and transforming growth factor-β1 (TGF-β1) mRNA expressions in a dose-dependent manner. Lipopolysacchorides (LPS) dramatically increased NF-κB DNA binding activity of HMCs, which was inhibited by IL-13 in a dose-dependent manner. LPS-activated NF-κB contained p50 and p65 dimers, but not c-Rel subunit. IL-13 blocked LPS-induced NF-κB subunit p65. LPS stimulated JNK/AP-1 activation, which was inhibited by IL-13 in a dose-dependent manner.

**Conclusion:**

IL-13 inhibits proinflammatory cytokines, chemokines, and profibrogenic cytokines synthesis by blocking NF-κB and JNK/AP-1 activation. These observations point to the importance of IL-13 in the modulation of inflammatory processes in the renal glomerulus.

## INTRODUCTION

The infiltration of glomeruli with macrophages and lymphocytes is a common pathological change observed in most cases of glomerulonephritis (GN). As these cells comprise a rich source of proinflammatory cytokines such as interleukin (IL) -1, IL-6, IL-8, and tumor necrosis factor-α (TNF-α), the role of these cytokines in the pathogenesis of GN has been extensively examined[1]–[3]. Despite numerous investigations of these proinflammatory factors, few studies have been undertaken on many other anti-inflammatory cytokines in connection with GN.

IL-13 is a novel lymphokine which was isolated from activated human T lymphocytes and confirmed to be expressed in a variety of cells[4]. It exhibits both anti-inflammatory and immuno-regulatory properties. The anti-inflammatory effect of IL-13 is achieved through the suppressed production of macrophage inflammatory proteins such as IL-1, IL-6, IL-8, IL-12, monocyte chemoattractant protein-1 (MCP-1), macrophage inflammatory protein-1 (MIP-1), and TNF-α, and through the enhanced production of IL-1 receptor antagonist[4],[5]. In addition, IL-13 inhibits protein kinase C -triggered respiratory bursts and suppresses nitric oxide release from macrophages and renal mesangial cells[6]. *In vivo*, IL-13 protects animals from lipopolysaccharides (LPS) -induced lethal endotoxemia and IgG immune complex-induced lung injury[7].

How IL-13 carries out its anti-inflammatory effects is not known. The nuclear transcription factors nuclear factor-kappa B (NF-κB) and activation protein-1 (AP-1) are known to play an important role in immune regulation and inflammation. These factors are present in an inactive state in the cytoplasm. When activated, they translocate to the nucleus, bind the DNA, and activate genes, such as IL-1, IL-6, IL-8, MCP-1, TNF-α, etc. A wide variety of inflammatory stimuli can activate NF-κB and AP-1, including TNF, IL-1, LPS, ceramide, and phorbol ester[8],[9].

*In vitro* studies from our laboratory and Yap *et al*[10] have shown that IL-13 expression was increased in peripheral blood mononuclear cells (PBMCs) in childhood nephrotic syndrome and in cultured mesangial cells. This stimulated us to investigate the effect of IL-13 on the proinflammatory cytokines, chemokines and profibrogenic cytokine expressions in mesangial cells and the involved molecular mechanisms. Our results showed that IL-13 inhibits LPS-induced TNF-α, IL-1α, IL-1β, MCP-1, IL-8, and TGF-β1 expressions in human mesangial cells (HMCs) by blocking the activation of NF-κB and c-Jun N-terminal kinase (JNK)/AP-1 pathway.

## MATERIALS AND METHODS

### Reagents

RPMI 1640, HEPES, fetal calf serum (FCS) and TRIzol reagent were purchased from Gibco BRL, USA. LPS from *Escherichia coli* 026:B6, insulin, transferrin and collagenase type IV were purchased from Sigma, USA. Cell culture flasks were purchased from Nunc, Denmark. Human multi-probe template sets were purchased from PharMingen, USA. Oligo (dT)_15_, dNTPs, AMV and Taq polymerase were purchased from Promega, USA. NF-κB, AP-1 and Specificity protein-1 (Sp-1) consensus oligonucleotide and rabbit polyclonal antibodies against NF-κB subunits p50, p65 and c-Rel were purchased from Santa Cruz Biotechnology, USA. Rabbit polyclonal anti-phospho-c-Jun (Ser63) antibody was purchased from Cell Signaling Technology Inc.,USA.

### Mesangial cells culture and treatments

Primary HMCs were isolated from normal-appearing portions of human kidneys that were surgically removed for renal carcinoma. HMCs were cultured as previously described[11]. Briefly, the cortex was separated from the medulla and minced, and glomeruli were isolated by a standard sieving technique through graded mesh screens (80, 100, 200 mesh). The glomerular suspension was collected, washed with RPMI 1640, and incubated with 2.5 mg/mL collagenase type IV at 37°C for 30 min. Isolated glumeruli were rinsed twice, resuspended in culture medium (RPMI 1640 buffered with 10 mmol/L HEPES to pH 7.4, and supplemented with 20% FCS, 5 µg/mL insulin and transferrin, 100 U/mL penicillin, and 100 mg/mL streptomycin) at 37°C in a humidified atmosphere of 5% CO_2_. Under these conditions, mesangial cells appeared after 4 d and reached confluency by d 7. Cells were confirmed as mesangial by morphological criteria, by the absence of staining for cytokeratin and von Willebrand factor, and by the presence of α-smooth muscle actin and vimentin. All experiments were performed using cells between passages 3 and 5.

The cells subcultured in 75-cm^2^ flasks at a density of 1×10^6^ cells/flask, were incubated in medium plus 20% FCS until approximately 80% confluence, and were then incubated in 0.5% FCS medium for 48 h to make the cells quiescent, when the studies were performed.

### RNA extraction

Total RNA of cultured HMCs was extracted by an acid guanidinium thiocyanate-phenol-chloroform extraction procedure using TRIzol reagent according to the manufacture's instruction. Extracted RNA was dissolved in 20-30 µL of diethylpyrocarbonate-treated water.

### Riboprobe synthesis

The multi-probe template set contains DNA templates, which can be used to transcribe radiolabeled antisense RNA probes, and can hybridize with target human mRNAs encoding TNF-α, IL-1α, IL-1β, MCP-1, IL-8, transforming growth factor-β1 (TGF-β1) and housekeeping gene product GAPDH. Riboprobes were transcribed by incubation with T7 polymerase and (α-^32^P) UTP according to the Ambion maxiscript T7 *in vitro* transcription protocol. The length of the ^32^P-labeled cRNA probes used are shown in ***Table 1***.

**Table 1:** The seven templates in the multi-probe template Set with corresponding unprotected and protected nucleotide sizes(nt)

Template	TNF-α	IL-1β	IL-lα	MCP-1	IL-8	TGF- βl	GAPDH
Unprotected probe	314	257	283	232	210	189	124
Protected probe	285	230	255	203	181	160	96

### Ribonuclease protection assay (RPA)

Ribonuclease protection assay was performed according to the protocol provided by Ambion (RPA III ribonuclease protection assay kit). Five times 10^5^ cpm of the cRNA probes were hybridized with 20 µg total RNA at 56°C overnight, and then digested with RNase A/T1. After phenol/chloroform extraction and ethanol precipitation, protected fragments were separated on a denaturing 10% polyacrylamide/urea sequencing gel and visualized by autoradiography at -80°C for 1-3 d. Results were quantified with an image analyzer (UVP). Densitometric quantitation of cytokines mRNA was expressed in arbitrary units as the ratio of cytokines/GAPDH.

In control experiments on hybridization specificity, 6.0 µg of yeast transfer RNA was hybridized and processed as described above. No signal corresponding to TNF-α, IL-1α, IL-1β, MCP-1, IL-8, TGF-β1 and GAPDH was detected.

### Electrophoretic mobility shift assay (EMSA)

At indicated time points, HMCs were rapidly washed with ice-cold PBS, pelleted (500 *g*, 10 min, 4°C), resuspended in hypotonic buffer (10 mmol/L HEPES, pH 7.9, 10 mmol/L KCl, 0.1 mmol/L EDTA, 0.1 mmol/L EGTA, 1 mmol/L DTT, 0.5 mmol/L PMSF, complete protease inhibitor cocktail tablets), and incubated for 10 min on ice. Cells were lysed by the addition of 0.1% Nonidet P-40. The nuclear pellet was collected by centrifugation (14,000 *g*, 10 min, 4°C), resuspended in extraction buffer (20 mmol/L HEPES, pH 7.9, 0.4 mmol/L KCl, 1 mmol/L EDTA, 1 mmol/L EGTA, 1 mmol/L DTT, 1 mmol/L PMSF, complete protease inhibit cocktail tablets) and incubated for 10 min on ice. The tubes were centrifuged again (14,000 *g*, 10 min, 4°C), and the supernatants were stored at -70°C until used. The protein content of the nuclear extracts was measured by the Bradford method (Bio-Rad Laboratories, USA).

EMSAs for NF-κB and AP-1 in nuclei were performed using gel shift assay system (Promega, USA). The reaction mixture contained 2 µL of 5× gel shift binding buffer, 1 µL of ^32^P-labeled double-stranded NF-κB or AP-1 consensus oligonucleotide probe, 2 µL of water, and 5 µL of nuclear extract (15 µg of protein). The binding reaction mixture was incubated at room temperature for 20 min and analyzed by electrophoresis on 5% non-denaturing polyacrylamide gel. After electrophoresis, the gels were dried by Gel-Drier (Bio-Rad Laboratories) and exposed to Kodak X-ray films at -70°C. An unlabeled double-stranded NF-κB or AP-1 consensus oligonucleotide and unrelated Sp-1 consensus oligonucleotide were used to examine the specificity of binding of NF-κB and AP-1 to the DNA. NF-κB/Rel proteins exist as dimers, therefore, supershift assays were performed to analyze the composition of activated NF-κB. In these studies, 1 µL of antibodies against specific NF-κB subunits p50, p65 and c-Rel protein was incubated with the reaction mixture for 30 min before the addition of radiolabeled NF-κB probes. Electrophoresis was performed as described, and the autoradiographs were analyzed for reductions in signal intensities and the presence of supershifted bands.

### Western blot analysis

At indicated time points, HMCs were rapidly washed with ice-cold PBS and lysed for 10 min on ice in lysis buffer (50 mmol/L Tris, pH 7.5, 40 mmol/L NaCl, 1% Triton X-100, 2 mmol/L EDTA, 1 µg/mL leupeptin, 2 mmol/L DTT, and 1 mmol/L PMSF). Lysates were cleared by centrifugation at 14,000 g (4°C) for 10 min. Total protein was quantified by the Bradford assay. Equal amounts of lysates were fractionated by 10% SDS-PAGE and electro-transferred onto Bio-Blot nitrocellulose membranes (Bio-Rad). Membranes were blocked for 1 h at room temperature in TBS-T (20 mmol/L Tris-base pH7.6, 150 mmol/L NaCl, 0.1% Tween-20) containing 5% bovine serum albumin, incubated with primary antibodies in the blocking solution at 4°C overnight. Subsequently, the membranes were incubated with HRP-conjugated secondary antibody (1:1,000) at room temperature for 1 h. Antibody binding was visualized with ECL kit (Amersham, USA), and quantitation of the chemiluminescent signal was carried out by UVP software.

### Statistical analysis

Each experiment was performed in triplicate. Measured values were expressed as mean±SD. Mean differences among groups were analyzed by one way analysis of variance (ANOVA). Student-Newman-Keuls (SNK) method was used for multiple comparisons. Statistical analyses were performed using SPSS software (Version 10.0, SPSS Inc., USA). *P* values < 0.05 were considered significant.

## RESULTS

### Effect of IL-13 on the cytokine mRNA expression

**Fig.1:**
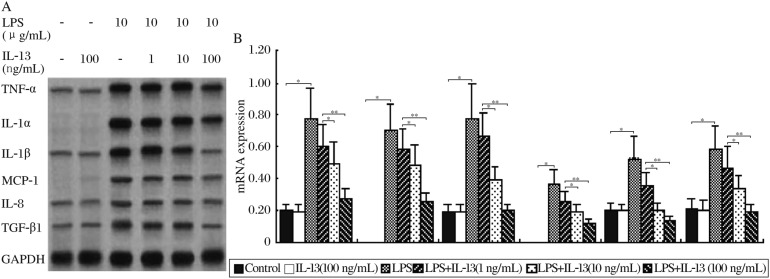
Inhibitory effect of IL-13 on the expression of proinflammatory cytokines, chemokines and profibrogentic cytokine specific mRNA levels by HMCs activated by LPS. A: HMCs were unstimulated or stimulated with LPS (10 µg/mL) for 12 h in the presence or absence of a 30-min pretreatment with different concentrations of IL-13 (1-100 ng/mL). After these treatments, total RNA was extracted and subjected to ribonuclease protection assay analysis for mRNA expression of TNF-α, IL-1α, IL-1β, MCP-1, IL-8 and TGF-β1. B: Densitometric quantitation of TNF-α, IL-1α, IL-1β, MCP-1, IL-8 and TGF-β1 mRNA was expressed in arbitrary units as the ratio of cytokines/GAPDH levels. Values represent mean±SD, *n* = 3 for each group (*P < 0.05, **P < 0.01).

To determine the effect of IL-13 on the levels of proinflammatory cytokines, chemokines and profibrogentic cytokines mRNA expressed by HMCs, HMCs were pretreated with various concentrations of recombinant human IL-13 for 30 min, and then were stimulated for 12 h with LPS. Cytokines mRNAs were analyzed by ribonuclease protection assay. HMCs incubated in medium alone did not express IL-1α and MCP-1 mRNA, and constitutive mRNA expression in unstimulated cells was found for TNF-α, IL-1β, IL-8 and TGF-β1. LPS significantly up-regulated TNF-α, IL-1α, IL-1β, MCP-1, IL-8 and TGF-β1 mRNA expression. IL-13 (100 ng/mL) on its own had no discernible effect on basal TNF-α mRNA level (***Fig.1***). However, when given in combination with LPS, we observed a dose-dependent inhibitory effect of IL-13 on the levels of TNF-α, IL-1α, IL-1β, MCP-1, IL-8 and TGF-β1 mRNAs (***Fig. 1***).

### Effect of IL-13 on LPS-induced NF-κB activation

**Fig.2:**
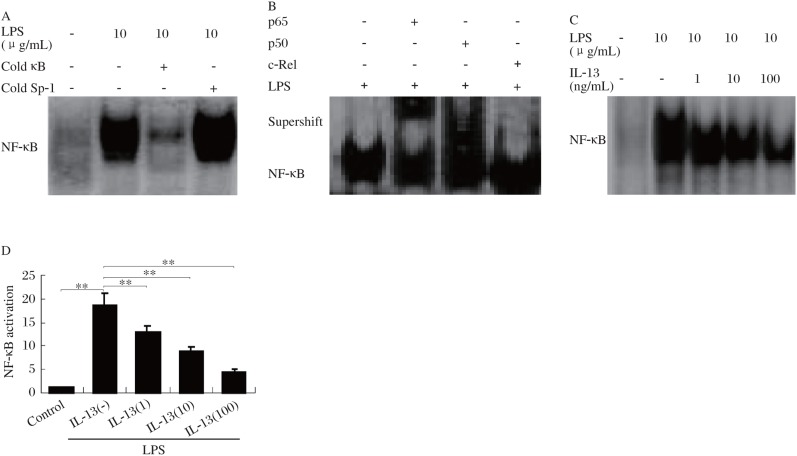
Effect of IL-13 on LPS-induced NF-κB activation. A. A competitional gel shift assay for NF-κB DNA-binding activity. EMSA with nuclear extracts from unstimulated or LPS-activated HMCs was carried out. 100-fold molar excess unrelated oligonucleotide containing the Sp-1 binding motif (cold Sp-1) did not inhibit binding of the radiolabeled NF-κB oligonucleotide, whereas 100-fold molar excess unlabeled NF-κB consensus sequence (cold κB) completely abrogated the signal. B: Supershift assay showed the interaction of anti-p65, anti-p50 and anti-cRel antibodies with NF-κB protein stimulated by LPS. C: HMCs were pretreated at 37°C for 30 min with different concentrations (0-100 ng/mL) of IL-13 followed by 1 h incubation with 10 µg/mL LPS. After these treatments, nuclear extracts were prepared and then assayed for NF-κB as described in materials and methods. D: Quantitation of EMSA blots by image analysis (C) was expressed in arbitrary units as the ratio of the control. Values represent mean±SD (*n* = 3, **P < 0.01).

Many of proinflammatory cytokines and chemokines that have been demonstrated to be suppressed by IL-13 are known to be regulated by the transcriptional factor NF-κB[12]. Therefore, the effect of IL-13 on the activation of NF-κB in HMCs was investigated in this study. As shown in ***Fig. 2A***, LPS dramatically increased NF-κB DNA binding activity when compared with the controls. To show that the retarded band visualized by EMSA in LPS-treated cells was NF-κB specific, extracts were mixed with excess cold NF-κB oligonucleotide, and irrelevant oligonucleotide (Sp-1 consensus oligonucleotide). The addition of excess cold NF-κB oligonucleotide (100-fold) resulted in complete absence of the band in the autoradiographs, and thus confirmed the specificity of the reaction (***Fig. 2A***). In contrast, the signal intensity was not affected by incubation with irrelevant Sp-1 oligonucleotide (***Fig. 2A***).

Supershift analysis was used to elucidate the composition of the activated NF-κB proteins. Nuclear extract from LPS-activated cells was incubated with antibody to either p50, p65 or c-Rel subunits, and then the EMSA was conducted. Incubation of extracts with antibodies against p50 and p65 significantly decreased the signal intensity and caused formation of a more slowly migrating, supershifted band (***Fig. 2B***), wherase anti-c-Rel antibody had no effect. This suggested that the LPS-activated NF-κB contained p50 and p65 dimers, but not c-Rel subunit (p50/p50 and p50/p65).

Pretreatment of IL-13 inhibited LPS-induced DNA binding in a dose-dependent manner (***Fig. 2C***). IL-13 at the concentration of 100 ng/mL decreased NF-κB activation by 80% (***Fig. 2D***).

### IL-13 inhibits LPS-induced NF-κB nuclear translocation

**Fig.3:**
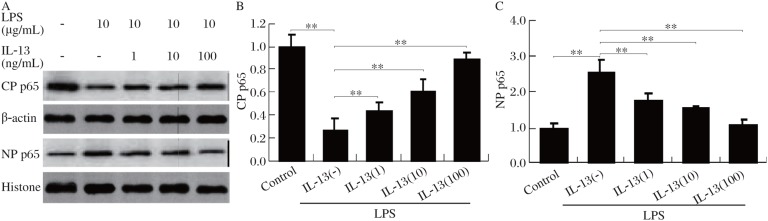
IL-13 inhibits LPS-induced NF-κB nuclear translocation. HMCs were pretreated for 30 min with different concentrations (0-100 ng/mL) of IL-13 followed by 1 h incubation with 10 µg/mL LPS. After these treatments, cytoplasmic protein (CP) and nuclear protein (NP) extracts were prepared and then assayed by western blot for p65 protein (A). Densitometry of the CP p65 (B) and NP p65 (C) immunoblot bands shown in A were expressed in arbitrary units as the ratio of the control. Values represent mean±SD (*n* = 3, **P < 0.01).

The translocation of NF-κB to the nucleus is preceded by phosphorylation and proteolytic degradation of IκB. Thus, one mechanism to explain the ability of IL-13 to inhibit NF-κB activity is by the ability of this anti-inflammatory cytokine to inhibit nuclear translocation of NF-κB in response to LPS stimulation. To address this question, cytoplasmic and nuclear proteins were isolated from LPS-stimulated HMCs, and immunoblot analysis was performed to determine the p65 protein. As shown in ***Fig. 3***, p65 was predominantly localized in the cytoplasm in unstimulated cells. Cells stimulated with LPS (30 min) demonstrated an increase in nuclear translocation of p65. In contrast, cells pretreated with IL-13 demonstrated a dose-dependent reduction in nuclear p65.

### Effect of IL-13 on LPS-induced AP-1 activation

**Fig.4:**
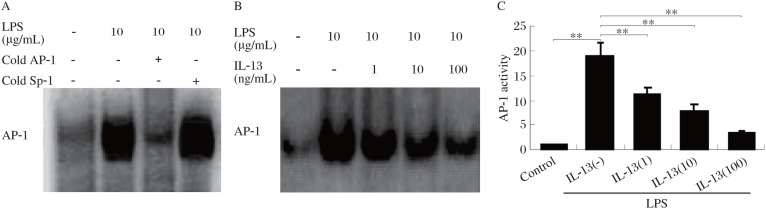
Effect of IL-13 on LPS-induced AP-1 activation. A: A competitioned gel shift assay for AP-1 DNA-binding activity. EMSA with nuclear extracts from unstimulated or LPS-activated HMCs was carried out. 100-fold molar excess unrelated oligonucleotide contatining the Sp1 binding motif (non-specific oligonucleotide) did not inhibit binding of the radiolabeled AP-1 oligonucleotide. 100-fold molar excess unlabeled AP-1 consensus sequence (specific competitor) completely abrogated the signal. The arrowhead (AP-1) indicates the AP-1-probe complexes. B: HMCs were pretreated at 37°C for 30 min with different concentrations (0-100 ng/mL) of IL-13 followed by 6 h incubation with 10 µg/mL LPS. After these treatments, nuclear extracts were prepared and then assayed for AP-1 as described in materials and methods. C: Quantitation of EMSA blots by image analysis (B) was expressed in arbitrary units as the ratio of the control. Values represent mean±SD (*n* = 3, **P < 0.01).

Besides NF-κB, LPS is a potent activator of another transcription factor, AP-1[13]. The mechanism of AP-1 activation, however, is known to be different from that of NF-κB[8],[9]. Thus, we also investigated the effect of IL-13 on LPS-induced AP-1 activation. Treatment with LPS induced a sevenfold increase in AP-1 binding. Pretreatment with IL-13 inhibited LPS-induced AP-1 activation in a dose-dependent manner (***Fig. 4***). Competition with the unlabeled AP-1 probe abolished DNA binding, indicating specificity. Thus, IL-13 also inhibited LPS-induced AP-1 activation, suggesting that IL-13 acts at a step common to both AP-1 and NF-κB activation.

### Effect of IL-13 on LPS-induced c-Jun N-terminal kinase (JNK) activation

**Fig.5:**
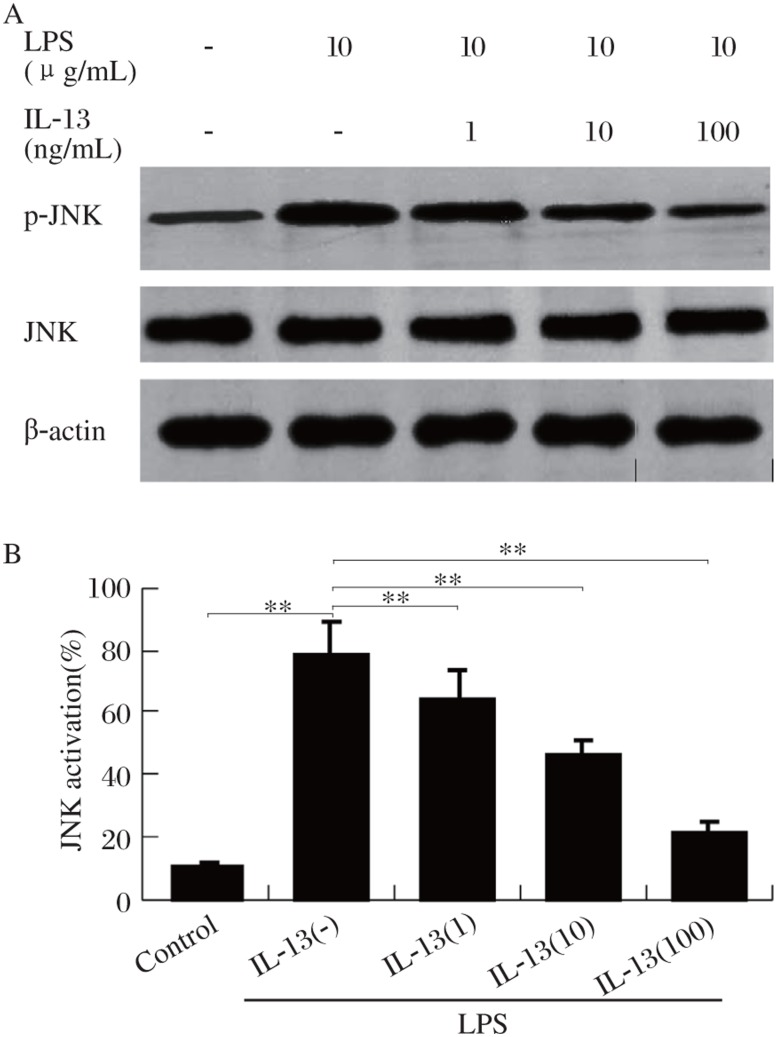
Effect of IL-13 on LPS-activated JNK. A: HMCs were pretreated at 37°C for 30 min with IL-13 (100 ng/mL), followed by LPS (10 µg/mL) for 60 min, and JNK activation was examined by immunoblot. B: Quantitation of blots by image analysis was expressed in arbitrary units as the ratio of the control. Values represent mean±SD. (*n* = 3, **P < 0.01).

A kinase that regulates AP-1 activation, JNK, is activated by LPS[14]. Therefore, we examined the effect of IL-13 on the activation of JNK. The exposure of HMCs to 10 µmol/L LPS for 30 min caused a 17-fold increase in the activation of JNK (***Fig. 5***). Pretreatment of cells with different concentrations of IL-13 significantly abolished LPS-induced JNK. Thus, it is possible that suppression of AP-1 activation by IL-13 is due to inhibition of JNK.

## DISCUSSION

Previous *in vitro* studies demonstrated that, upon stimulation with LPS or immune complexes, mesangial cells express genes and secrete newly synthesized IL-1, IL-6, IL-8, TNF-α and colony-stimulating factors. These cytokines have a critical role in amplifying the glomerular inflammatory reaction.

IL-13 is a recently characterized cytokine involved in the control of the inflammatory response, mainly because of its ability to suppress the synthesis of proinflammatory mediators by monocytes/macrophages. *In vitro*, IL-13 prevents production of proinflammatory cytokines by activating macrophages and monocytes. It has been shown that IL-13 potently inhibits the expression of the inducible isoform of nitric oxide synthase (iNOS) and cyclooxygenase-2 and prostaglandin E_2_ production by mesangial cells[6],[15], and inhibits vascular permeability factor release by peripheral blood mouonuclear cells (PBMCs) from patients with lipoid nephrosis[16]. *In vivo*, IL-13 has been shown to protect against LPS-induced lethality, and to suppress lung inflammatory injury following deposition of IgG immune complexes, and to inhibit liver injury induced by ischemia/reperfusion[17]. In these models, the protective effects of IL-13 were associated with reduced production of proinflammatory cytokines. In the current study, we showed that human IL-13 strongly inhibited IL-1α, IL-1β, TNF-α, IL-8, MCP-1 and TGF-β1 synthesis by HMCs following activation with LPS in a dose-dependent manner. Inhibition of the synthesis of these cytokines occurred at the transcriptional level. IL-13 added at a concentration of 100 ng/mL reduced IL-1β, IL-8, MCP-1 and TGF-β1 synthesis by more than 80% following activation of HMCs stimulated by LPS. The inhibitory effects on IL-1α and TNF-α synthesis were somewhat less pronounced. The results herein described are in agreement with the negative modulatory effect of IL-13 on the synthesis of IL-1 and TNF observed in human monocytes[18]. However, an opposite, stimulative effect of IL-13 on IL-8 and MCP-1 expression has been described in bronchial epithelial cells[19],[20]. In addition a recent report has shown that IL-13 failed to suppress MCP-1 secretion in myelomonocytic cells[21]. These observations suggest that cell type specificity is a critical issue to consider when interpreting effects promoted by LPS exposure.

The use of systemic IL-13 gene therapy may be useful in reducing renal inflammation caused by ischemia-reperfusion by diminishing the expression of E-selectin, IL-8, MIP-2, TNF-α and MCP-1 mRNA *in vivo*[22]. These findings are in accordance with the above observation that IL-13 significantly inhibited proinflammatory cytokines, chemokines and profibrogenic cytokine synthesis. Also, IL-13 mRNA has been detected from glomeruli of rats in the heterologous phase of nephrotoxic nephritis (NTN) [23]. Our studies and those of Yap *et al*[10] have shown that IL-13 expression was increased in PBMCs in childhood nephrotic syndrome and in cultured mesangial cells. These data suggested that IL-13 was expressed in glomeruli after the induction of immune or nonimmune injury and that, in turn, IL-13 was responsible for a regulatory feedback loop.

Inhibitory effects of IL-13 on proinflammatory cytokines/chemokines expression are of potential importance because these proinflammatory cytokines/chemokines contribute to glomerular injury in numerous experimental models of GN and human GN. This has been suggested by the observation that administration of IL-1 and TNF increased the severity of injury in the heterologous phase of NTN, and administration of IL-1 receptor antagonist (IL-1ra) or soluble IL-1 or TNF receptors or anti-IL-8 antibody provided protection, and administration of anti-MCP-1 monoclonal antibody significantly suppressed glomerular monocyte/macrophage infiltration and reduced urinary protein excretion in rats treated with nephrotoxic serum[24]–[26].

TGF-β is a well-known multifunctional cytokine produced by a variety of cell types: monocytes, macrophages, lymphocytes, fibroblasts, endothelial cells and mesangial cells. Previous studies have demonstrated an important role for TGF-β1 in renal disease in that TGF-β1 appeared to be the major cytokine that regulated the cell proliferation, differentiation, and angiogenesis, increased the synthesis of matrix proteins such as fibronectin and collagen types I and III, decreased synthesis of proteases which could digest matrix, and mediated expansion of extracellular matrix and fibrosis in various models[27]–[29]. TGF-β1 was also strongly implicated in the inflammatory process[30]. Released at the site of inflammation, TGF-β1 promoted leukocyte recruitment, including monocytes and neutrophils, as well as the activation and cytokine synthesis by these cells. In the present study, we demonstrated that IL-13 strongly inhibited TGF-β1 mRNA expression in a dose-dependent manner. It has been reported that IL-13 strongly inhibited proinflammatory cytokine-induced fibronectin production. In addition, IL-1α-stimulated type I and IV collagen synthesis was suppressed by concomitant IL-4 treatment in cultured HMCs. IL-4 and IL-13 have similar ranges of activities and bind to receptors sharring some of the same components[31]. On the basis of the work reported herein, the possibility should be considered that IL-13 might have antifibrogenic properties by downregulating profibrogenic cytokines, like TGF-β1 and collagen synthesis. However, the antifibrogenic properties of IL-13 require additional examination and will be the subject of further reports.

The mechanism by which IL-13 inhibits cytokines production by HMC is not clear. The IL-1, TNF, IL-8, MCP-1 and TGF-β promoter region has been partially characterized, and the activation of the transcriptional factor NF-κB and AP-1 appears to be necessary for induction of these cytokines in diverse cell types[32],[33]. In agreement with previous studies[12],[34], our results also showed that IL-13 inhibited LPS-mediated NF-κB and AP-1 activation. NF-κB is considered an important transcriptional regulator that plays a pivotal role in the immune response because of its regulation of the cytokines expression[35]. Normally, NF-κB is retained in the cytoplasm by binding to an inhibitor protein, IκB. Upon stimulation, IκB is phosphorylated and degraded, and separated from NF-κB. Then the activated NF-κB is translocated into the nucleus to active transcriptional expression of downstream genes associated with inflammatory responses[9]. In the present study, we found that IL-13 hindered NF-κB activation by inhibiting LPS-induced NF-κB subunit p65 nuclear translocation. Moreover, through results from EMSA, we demonstrated that IL-13 prevented LPS-induced DNA binding activity. In addition to the NF-κB activation, we showed that IL-13 inhibited AP-1 activation and blocked JNK phosphorylation by LPS. It has been shown that AP-1 is a transcription factor regulating the production of LPS-induced proinflammatory factors that can be regulated by JNK phosphorylation[12]. So it is possible that suppression of AP-1 by IL-13 is correlated with inhibition of JNK. All these observations indicate that the inhibitory effects of IL-13 on the activation of these cytokine promoters could be related to suppression of NF-κB and JNK/AP-1 activation.

In summary, the data obtained in this study demonstrate that IL-13 inhibits proinflammatory cytokines, chemokines, and profibrogenic cytokine synthesis by blocking the activation of NF-κB and JNK/AP-1. These observations point to the importance of this cytokine in the modulation of inflammatory processes in the renal glomerulus.

## References

[1] Karkar A (2008). Modulation of renal inflammation: therapeutic strategies. Saudi J Kidney Dis Transpl.

[2] Lan HY (2008). Role of macrophage migration inhibition factor in kidney disease. Nephron Exp Nephrol.

[3] Ernandez T, Mayadas TN (2009). Immunoregulatory role of TNFalpha in inflammatory kidney diseases. Kidney Int.

[4] Minty A, Chalon P, Derocq JM, Dumont X, Guillemot JC, Kaghad M (1993). Interleukin-13 is a new human lymphokine regulating inflammatory and immune responses. Nature.

[5] Sikora JP, Kuzanski W, Andrzejewska E (2009). Soluble cytokine receptors sTNFR I and sTNFR II, receptor antagonist IL-1ra, and anti-inflammatory cytokines IL-10 and IL-13 in the pathogenesis of systemic inflammatory response syndrome in the course of burns in children. Med Sci Monit.

[6] Saura M, Martinez-Dalmau R, Minty A, Perez-Sala D, Lamas S (1996). Interleukin-13 inhibits inducible nitric oxide synthase expression in human mesangial cells. Biochem J.

[7] Lentsch AB, Shanley TP, Sarma V, Ward PA (1997). *In vivo* suppression of NF-kappa B and preservation of I kappa B alpha by interleukin-10 and interleukin-13. J Clin Invest.

[8] Herbein G, Varin A, Fulop T (2006). NF-kappaB, AP-1, Zinc-deficiency and aging. Biogerontology.

[9] Maziere C, Maziere JC (2009). Activation of transcription factors and gene expression by oxidized low-density lipoprotein. Free Radic Biol Med.

[10] Yap HK, Cheung W, Murugasu B, Sim SK, Seah CC, Jordan SC (1999). Th1 and Th2 cytokine mRNA profiles in childhood nephrotic syndrome: evidence for increased IL-13 mRNA expression in relapse. J Am Soc Nephrol.

[11] Di Marco GS, Naffah-Mazzacoratti Md Mda G, Vio CP, Dos Santos OF, Schor N, Casarini DE (2003). Mesangial cells are able to produce catecholamines *in vitro*. J Cell Biochem.

[12] Manna SK, Aggarwal BB (1998). IL-13 suppresses TNF-induced activation of nuclear factor-kappa B, activation protein-1, and apoptosis. J Immunol.

[13] Lin WN, Luo SF, Lin CC, Hsiao LD, Yang CM (2009). Differential involvement of PKC-dependent MAPKs activation in lipopolysaccharide-induced AP-1 expression in human tracheal smooth muscle cells. Cell Signal.

[14] Hui X, Li H, Zhou Z, Lam KS, Xiao Y, Wu D (2010). Adipocyte fatty acid-binding protein modulates inflammatory responses in macrophages through a positive feedback loop involving c-Jun NH2-terminal kinases and activator protein-1. J Biol Chem.

[15] Elnaggar R, Hanawa H, Liu H, Yoshida T, Hayashi M, Watanabe R (2005). The effect of hydrodynamics-based delivery of an IL-13-Ig fusion gene for experimental autoimmune myocarditis in rats and its possible mechanism. Eur J Immunol.

[16] Matsumoto K, Ohi H, Kanmatsuse K (1997). Interleukin 10 and interleukin 13 synergize to inhibit vascular permeability factor release by peripheral blood mononuclear cells from patients with lipoid nephrosis. Nephron.

[17] Kato A, Okaya T, Lentsch AB (2003). Endogenous IL-13 protects hepatocytes and vascular endothelial cells during ischemia/reperfusion injury. Hepatology.

[18] de Waal Malefyt R, Figdor CG, Huijbens R, Mohan-Peterson S, Bennett B, Culpepper J (1993). Effects of IL-13 on phenotype, cytokine production, and cytotoxic function of human monocytes. Comparison with IL-4 and modulation by IFN-gamma or IL-10. J Immunol.

[19] Striz I, Mio T, Adachi Y, Robbins RA, Romberger DJ, Rennard SI (1999). IL-4 and IL-13 stimulate human bronchial epithelial cells to release IL-8. Inflammation.

[20] Gu N, Kang G, Jin C, Xu Y, Zhang Z, Erle DJ (1999). Intelectin is required for IL-13-induced monocyte chemotactic protein-1 and -3 expression in lung epithelial cells and promotes allergic airway inflammation. Am J Physiol Lung Cell Mol Physiol.

[21] Steube KG, Meyer C, Drexler HG (1999). Constitutive protein expression of monocyte chemotactic protein-1 (MCP-1) by myelomonocytic cell lines and regulation of the secretion by anti- and proinflammatory stimuli. Leuk Res.

[22] Sandovici M, Henning RH, van Goor H, Helfrich W, de Zeeuw D, Deelman LE (2008). Systemic gene therapy with interleukin-13 attenuates renal ischemia-reperfusion injury. Kidney Int.

[23] Tipping PG, Kitching AR (2005). Glomerulonephritis, Th1 and Th2: what's new?. Clin Exp Immunol.

[24] Karkar AM, Tam FW, Steinkasserer A, Kurrle R, Langner K, Scallon BJ (1995). Modulation of antibody-mediated glomerular injury *in vivo* by IL-1ra, soluble IL-1 receptor, and soluble TNF receptor. Kidney Int.

[25] Rusai K, Huang H, Sayed N, Strobl M, Roos M, Schmaderer C (2008). Administration of interleukin-1 receptor antagonist ameliorates renal ischemia-reperfusion injury. Transpl Int.

[26] Fujinaka H, Yamamoto T, Takeya M, Feng L, Kawasaki K, Yaoita E (1997). Suppression of anti-glomerular basement membrane nephritis by administration of anti-monocyte chemoattractant protein-1 antibody in WKY rats. J Am Soc Nephrol.

[27] Rawlins JT, Opperman LA (2008). Tgf-beta regulation of suture morphogenesis and growth. Front Oral Biol.

[28] Omwandho CO, Konrad L, Halis G, Oehmke F, Tinneberg HR Role of TGF-betas in normal human endometrium and endometriosis. Hum Reprod.

[29] Schnaper HW, Jandeska S, Runyan CE, Hubchak SC, Basu RK, Curley JF (2009). TGF-beta signal transduction in chronic kidney disease. Front Biosci.

[30] Sanjabi S, Zenewicz LA, Kamanaka M, Flavell RA (2009). Anti-inflammatory and pro-inflammatory roles of TGF-beta, IL-10, and IL-22 in immunity and autoimmunity. Curr Opin Pharmacol.

[31] Andrews AL, Nordgren IK, Kirby I, Holloway JW, Holgate ST, Davies DE (2009). Cytoplasmic tail of IL-13Ralpha2 regulates IL-4 signal transduction. Biochem Soc Trans.

[32] Shinozaki S, Mashima H, Ohnishi H, Sugano K (2010). IL-13 promotes the proliferation of rat pancreatic stellate cells through the suppression of NF-kappaB/TGF-beta1 pathway. Biochem Biophys Res Commun.

[33] Lan Y, Zhou Q, Wu ZL (2004). NF-kappa B involved in transcription enhancement of TGF-beta 1 induced by Ox-LDL in rat mesangial cells. Chin Med J (Engl).

[34] Deepak P, Kumar S, Acharya A (2007). Interleukin-13-induced type II polarization of inflammatory macrophages is mediated through suppression of nuclear factor-kappaB and preservation of IkappaBalpha in a T cell lymphoma. Clin Exp Immunol.

[35] Li X, Stark GR (2002). NFkappaB-dependent signaling pathways. Exp Hematol.

